# Supplementary material for: Bronchial Physiological Saline Injection to Visualize Peripheral Pulmonary Lesions in Ultrathin Bronchoscopy
Source: Diagnostics (Basel). 2025 Nov 28;15(23):3029. doi: 10.3390/diagnostics15233029 (PMC12691501; doi:10.3390/diagnostics15233029)
Supplement: Supplementary file 1 [file diagnostics-15-03029-s001.zip › e-Figure_legends20250922.pdf]

Title

Bronchial physiological saline injection to visualize peripheral pulmonary lesions in ultrathin bronchoscopy

Authors

Mika Nakao[1], Tamio Okimoto[1], Noriaki Kurimoto[1], Ryosuke Tanino[1], Misato Kobayashi[1], Kazuhisa Nakashima[1], Takamasa Hotta[1], Yukari Tsubata[1], Takeshi Isobe[1]

Online Data Supplement

Figure legends

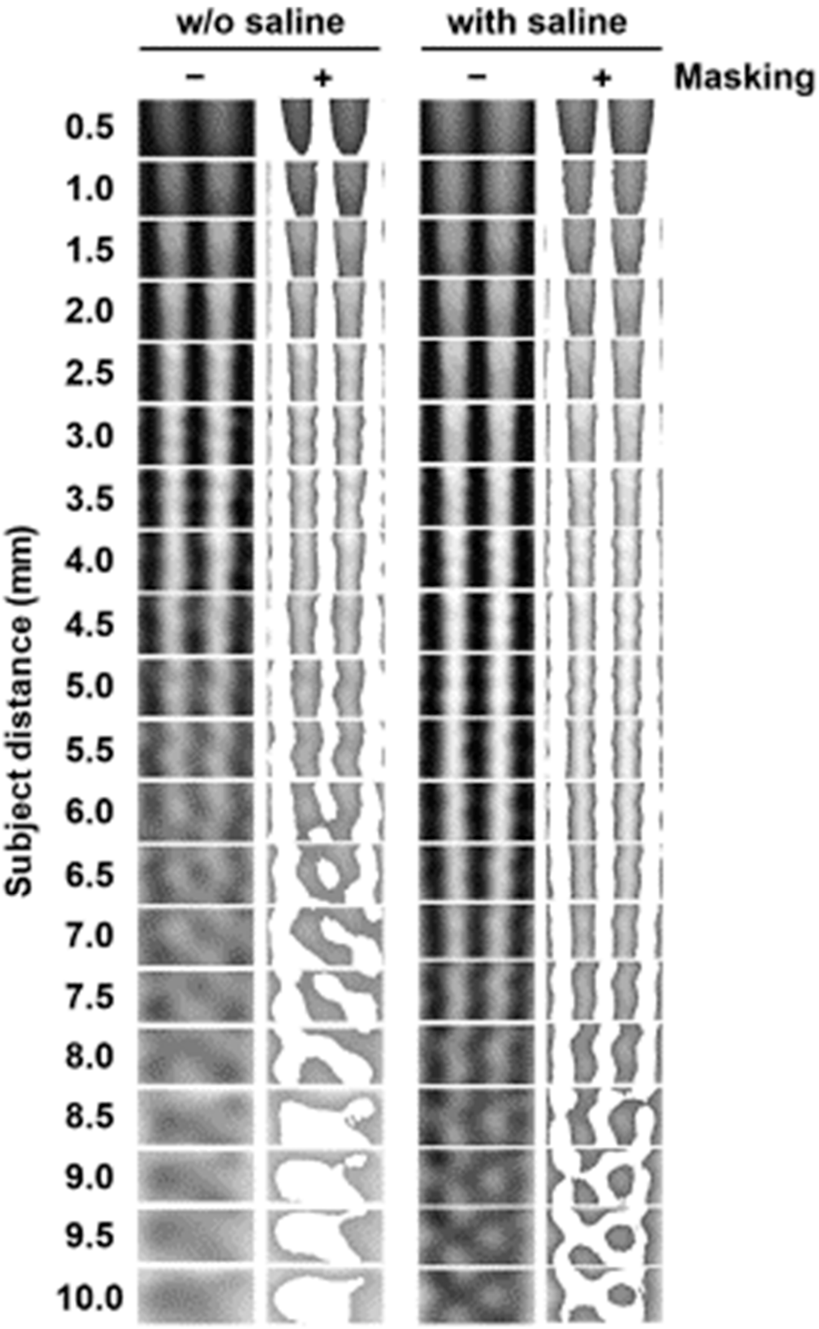

Figure S1. Saline increased the range of subject distance with separated white mask areas in the evaluation images obtained using narrow-band imaging.

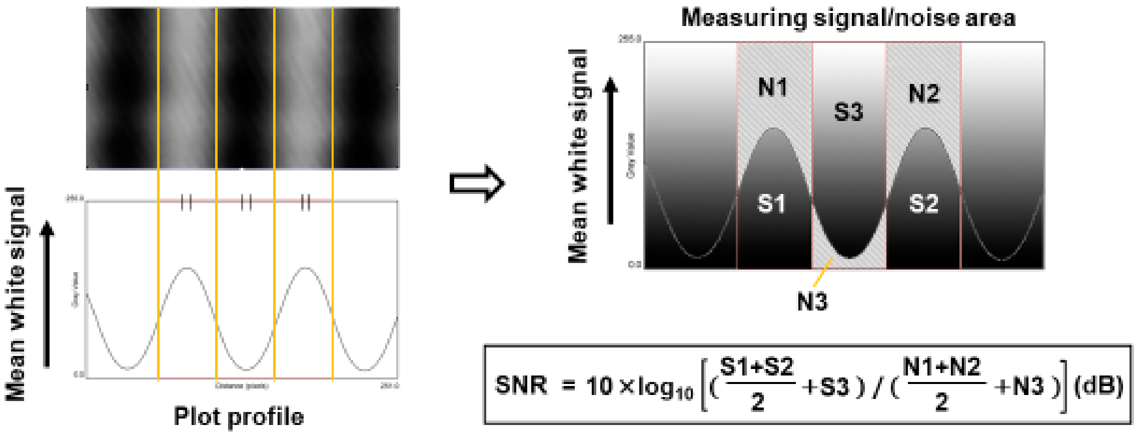

Figure S2. Schematic diagram of the procedure for measuring signal-to-noise ratio (SNR).
