# Supplementary material for: Bronchial Physiological Saline Injection to Visualize Peripheral Pulmonary Lesions in Ultrathin Bronchoscopy
Source: Diagnostics (Basel). 2025 Nov 28;15(23):3029. doi: 10.3390/diagnostics15233029 (PMC12691501; doi:10.3390/diagnostics15233029)
Supplement: Supplementary file 1 [file diagnostics-15-03029-s001.zip › e-Table_1_20250927.pdf]

Title

Bronchial physiological saline injection to visualize peripheral pulmonary lesions in ultrathin bronchoscopy

Authors

Mika Nakao[1], Tamio Okimoto[1], Noriaki Kurimoto[1], Ryosuke Tanino[1], Misato Kobayashi[1], Kazuhisa Nakashima[1], Takamasa Hotta[1], Yukari Tsubata[1], Takeshi Isobe[1]

Online Data Supplement

Table S1. Ultrathin bronchoscopic findings of raters 1 and 2 following air or saline injection in narrow-band imaging

		%Distinguishable cases		*P* value (exact McNemar test)
		Air	Saline	
Rater 1	The unevenness of the bronchial luminal surface	81.8	100.0	0.5
	Circular and/or longitudinal folds	81.8	100.0	0.5
	Blood vessels of the subepithelium	36.4	100.0	0.016
	Perspective	36.4	100.0	0.016
Rater 2	The unevenness of the bronchial luminal surface	18.2	36.4	0.63
	Circular and/or longitudinal folds	72.7	72.7	1
	Blood vessels of the subepithelium	36.4	45.5	1
	Perspective	81.8	72.7	1
